# Supplementary material for: Encapsulation of Metronidazole in Biocompatible Macrocycles and Structural Characterization of Its Nano Spray-Dried Nanostructured Composite
Source: Molecules. 2021 Dec 2;26(23):7335. doi: 10.3390/molecules26237335 (PMC8659152; doi:10.3390/molecules26237335)
Supplement: Supplementary file 1 [file molecules-26-07335-s001.zip › molecules-1480026-supplementary.pdf]

Encapsulation of metronidazole in biocompatible macrocycles and structural characterization of its nano spray-dried nanostructured composite

Mirella Mirankó [1], Mónika Megyesi [2], Zsombor Miskolczy [2], Judit Tóth [1,2], Tivadar Feczkó [1,2,]* and László Biczók [2,]*

[1] University of Pannonia, Faculty of Engineering, Research Institute of Biomolecular and Chemical Engineering, 8200 Veszprém, Egyetem u. 10., Hungary; miranko@mukki.richem.hu (Mi.M); toth@mukki.richem.hu (J.T); feczko@mukki.richem.hu (T.F.)

[2] Research Centre for Natural Sciences, Institute of Materials and Environmental Chemistry, Eötvös Loránd Research Network (ELKH), P.O. Box 286, 1519 Budapest, Hungary; megyesi.monika@ttk.hu (Mó.M.); miskolczy.zsombor@ttk.hu (Z.M.); biczok.laszlo@ttk.hu (L.B.)

***** Correspondence: feczko@mukki.richem.hu (T.F.); biczok.laszlo@ttk.hu (L.B.)

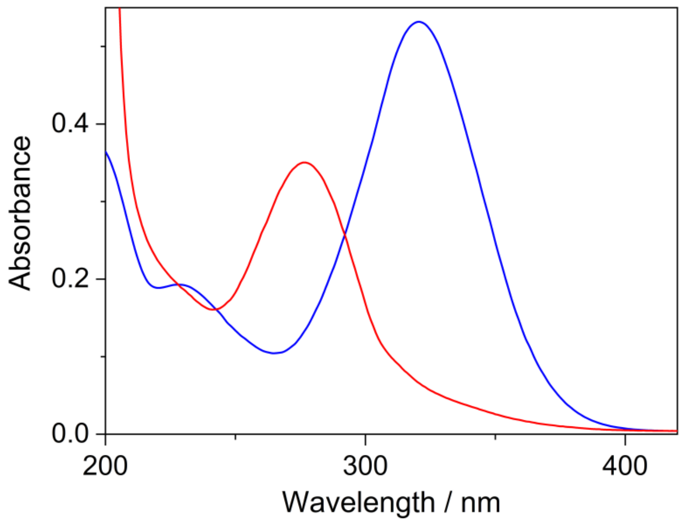

Figure S1 Absorption spectra of metronidazole in water (blue) and in 0.1 N H_2SO_4 solution (red)

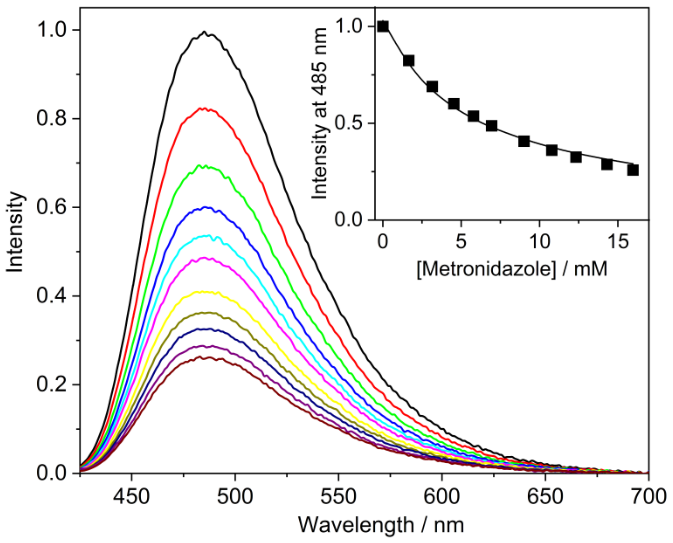

Figure S2 Alteration of the fluorescence spectra in the aqueous solution of 1.0 μM dehydrocorydaline and 3.0 μM CB7 at 0, 1.7, 3.2, 4.5, 5.8, 6.9, 9.0, 10.8, 12.3, 14.3, and 16.0 mM metronidazole concentrations (pH = 7.5, excitation wavelength 420 nm). Inset: fluorescence intensity at 485 nm as a function of metronidazole concentration.

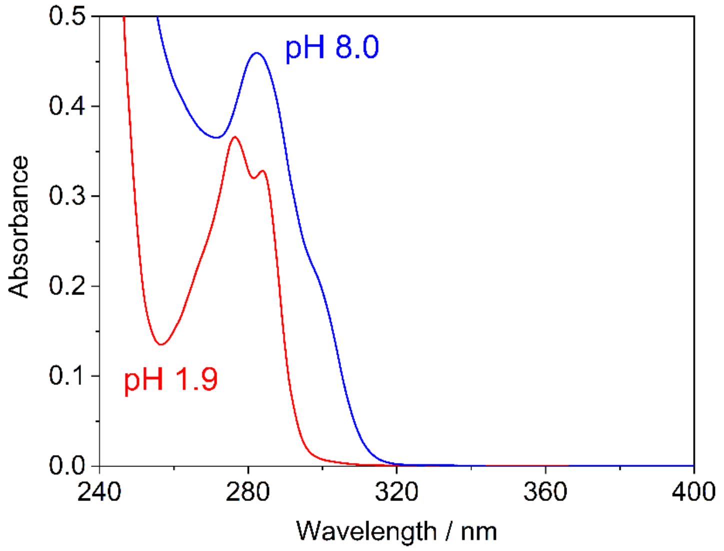

Figure S3 Absorption spectra of 80 μM 4-sulfonatocalix[4]arene (SCX4) in water at pHs 1.9 and 8.0

Evaluation of the binding constant of $MetH^+$ confinement in CB7

Protonation of CB7 is also an unimportant process at pH 3 because it has an equilibrium constant of only ~166 M^{-1} [1]. The mass balance equations are:

$$[MetH^+ - CB7] + [Met - CB7] + [MetH^+] + [Met] = [Met]_T \quad (S1)$$

$$[MetH^+ - CB7] + [Met - CB7] + [CB7] = [CB7]_T \quad (S2)$$

The measurements were performed at equal total concentrations of metronidazole and CB7, $[CB7]_T = [Met]_T$. Therefore, (S2) and (S1) gives

$$[MetH^+] + [Met] = [CB7] \quad (S3)$$

Based on the definition of the equilibrium constant of the proton dissociation from $MetH^+$ (K_a), the following relationship is derived:

$$[MetH^+] = \frac{[Met][H^+]}{K_a} \quad (S4)$$

Substitution (S4) into (S3) gives

$$[Met]\left(1 + \frac{[H^+]}{K_a}\right) = [CB7] \quad (S5)$$

The definition of the equilibrium constant of $MetH^+$–CB7 complex formation (K_p) gives:

$$[MetH^+ - CB7] = K_p[MetH^+][CB7] \quad (S6)$$

Substitution (S4) into (S6) results in

$$[MetH^+ - CB7] = K_p \frac{[Met][H^+]}{K_a}[CB7] \quad (S7)$$

Substitution (S5) into (S7) leads to

$$[MetH^+ - CB7] = K_p \frac{[H^+]}{K_a}\left(1 + \frac{[H^+]}{K_a}\right)[Met]^2 \quad (S8)$$

The definition of the equilibrium constant of Met–CB7 complex formation (K) gives:

$$[Met - CB7] = K[Met][CB7] \quad (S9)$$

Substitution (S5) into (S9) provides

$$[Met - CB7] = K\left(1 + \frac{[H^+]}{K_a}\right)[Met]^2 \quad (S10)$$

Substitution (S5), (S8), and (S10) into (S2) and rearrangement leads to

$$\left(K + K_p \frac{[H^+]}{K_a}\right)\left(1 + \frac{[H^+]}{K_a}\right)[Met]^2 + \left(1 + \frac{[H^+]}{K_a}\right)[Met] - [CB7]_T = 0 \qquad \text{(S11)}$$

where K_p is a fitting parameter, $[H^+] = 10^{-3}$ M, $K_a = 4.365\times10^{-3}$ M was determined by spectrophotometric titration, and K=1000 M^{-1} based on the experiments with dehydrocorydaline fluorescent probe.

$$q = \left(1 + \frac{[H^+]}{K_a}\right) = 1.2291 \qquad \text{(S12)}$$

The solution of (S11) quadratic equation using (S12) provides [Met] at each $[CB7]_T$ concentration.

$$[Met] = \frac{-q + \sqrt{q^2 + 4\left(K + K_p[H^+]/K_a\right)q[CB7]_T}}{2\left(K + K_p[H^+]/K_a\right)q} \qquad \text{(S13)}$$

Using these values, $[MetH^+]$, [Met–CB7] and $[MetH^+–CB7]$ can be calculated by (S4), (S10), and (S8). In our experiments, the apparent molar absorption coefficient (ε) was measured at 320 nm as a function of the total CB7 concentration. The result is expressed by the following relationship:

$$\varepsilon = \varepsilon_1 \frac{[Met]}{[Met]_T} + \varepsilon_2 \frac{[MetH^+]}{[Met]_T} + \varepsilon_3 \frac{[Met-CB7]}{[Met]_T} + \varepsilon_4 \frac{[MetH^+-CB7]}{[Met]_T} \qquad \text{(S14)}$$

where ε_1, ε_2, ε_3, and ε_4 represent the molar absorption coefficient of Met, $MetH^+$, Met–CB7, and $MetH^+$–CB7, respectively. Independent experiments provided ε_1 = 8730 M^{-1} cm^{-1}, ε_2 = 780 M^{-1} cm^{-1}, K = 1000 M^{-1}, which were kept constant in the nonlinear least-squares analysis of the experimental data. Starting with the initial estimates of K_p, ε_3 and ε_4, we calculated [Met], $[MetH^+]$, [Met–CB7], $[MetH^+–CB7]$ and ε by (S13), (S4), (S10), (S8) and (S14), respectively. Then, the iterations were repeated until the best fit of ε was achieved. The optimized function gave $K_p = 1.9\times10^5$ M^{-1}, ε_3 = 8800 M^{-1} cm^{-1} and ε_4 = 620 M^{-1} cm^{-1} values.

Evaluation of the binding constant of MetH$^+$ confinement in SCX4

Berberine (B) was used as a fluorescent probe for the determination of the binding constant of MetH$^+$ association with SCX4. Since the total concentration of B ($[B]_T$ = 5.0 μM) was much lower than the total SCX4 concentration ($[SCX4]_T$ = 243 μM), B-SCX4 complex can be neglected in the mass balance equations.

$$[SCX4]_T = [SCX4] + [Met - SCX4] + [MetH^+ - SCX4] \quad \text{(S15)}$$

$$[Met]_T = [Met] + [MetH^+] + [Met - SCX4] + [MetH^+ - SCX4] \quad \text{(S16)}$$

(S15) and (S16) lead to

$$[Met]_T - [SCX4]_T = [Met] + [MetH^+] - [SCX4] \quad \text{(S17)}$$

Based on the definition of the equilibrium constant of the proton dissociation from MetH$^+$ (K_a) in water we obtain

$$[Met] = \frac{[MetH^+]K_a}{[H^+]} \quad \text{(S18)}$$

(S17) and (S18) give

$$[Met]_T - [SCX4]_T = \left(\frac{K_a}{[H^+]} + 1\right)[MetH^+] - [SCX4] \quad \text{(S19)}$$

We define the following constant

$$a = \frac{[H^+]}{K_a + [H^+]} \quad \text{(S20)}$$

Substitution of (S20) into (S19) provides

$$[MetH^+] = a(\,[Met]_T - [SCX4]_T + [SCX4]) \quad \text{(S21)}$$

The definition of the equilibrium constant of Met–SCX4 and MetH$^+$–SCX4 complex formation (K and K_p) gives

$$[Met - SCX4] = K[SCX4][Met] \quad \text{(S22)}$$

$$[MetH^+ - SCX4] = K_p[SCX4][MetH^+] \quad \text{(S23)}$$

Substitution (S18) into (S22) gives

$$[Met - SCX4] = K[SCX4]\frac{[MetH^{+}]K_a}{[H^{+}]} \quad \text{(S24)}$$

Substitution (S23) and (S24) into (S15) results in

$$[SCX4]_T = [SCX4] + K[SCX4]\frac{[MetH^{+}]K_a}{[H^{+}]} + K_p[SCX4][MetH^{+}] \quad \text{(S25)}$$

Substitution (S21) into (S25) provides

$$[SCX4]_T = [SCX4]\left\{1 + \left(\frac{KK_a}{[H^{+}]} + K_p\right)a(\,[Met]_T - [SCX4]_T + [SCX4])\right\} \quad \text{(S26)}$$

The following quantity is defined:

$$b = \frac{KK_a}{[H^{+}]} + K_p \quad \text{(S27)}$$

(S27) and (S26) lead to

$$0 = -[SCX4]_T + [SCX4]\{1 + ba(\,[Met]_T - [SCX4]_T)\} + b[SCX4]^2 \quad \text{(S28)}$$

The solution of the quadratic equation is

$$[SCX4] = \frac{-\{1+ba(\,[Met]_T-[SCX4]_T)\}+\sqrt{\{1+ba(\,[Met]_T-[SCX4]_T)\}^2+4b[SCX4]_T}}{2b} \quad \text{(S29)}$$

The total concentration of berberine fluorescent probe is the sum of its unbound and SCX4-complexed fraction.

$$[B]_T = [B] + [B - SCX4] \quad \text{(S30)}$$

Using (S30) and the definition of the binding constant of B–SCX4 complex formation (K_B = 3720 M^{-1}) [2], the following relationship is obtained:

$$\frac{[B-SCX4]}{[B]_T} = \frac{K_B[SCX4]}{1+K_B[SCX4]} \quad \text{(S31)}$$

The fluorescence intensity (*I*) is proportional to the contribution arising from the fraction of the SCX4-complexed and free berberine:

$$I = \varphi_1\frac{K_B[SCX4]}{1+K_B[SCX4]} + \varphi_2\frac{1}{1+K_B[SCX4]} \quad \text{(S32)}$$

where φ_1 and φ_2 parameters are related to the efficiency of fluorescence emission for complexed and free berberine. The fluorescence quantum yields are 0.018 for B–SCX4 complex at pH 2 [2] and 0.00047 for free B in D_2O [3]. Therefore, the following relationship is employed:

$$\varphi_2 = \frac{0.00047}{0.018}\varphi_1 = 0.026\varphi_1 \quad \text{(S33)}$$

[SCX4] is calculated by (S29) and φ_1, K, and K_p parameters were optimised using $[H^+] = 0.01$ M and $K_a = 4.365\times10^{-3}$ M. The latter quantity was obtained from the $pK_a = 2.36$ value of $MetH^+$ deprotonation in water determined by spectrophotometric titrations.

References

[1] S. Zhang, L. Grimm, Z. Miskolczy, L. Biczók, F. Biedermann, W.M. Nau, Binding affinities of cucurbit[n]urils towards cations, Chem. Commun. 55 (2019) 14131-14134.

[2] M. Megyesi, L. Biczók, Considerable fluorescence enhancement upon supramolecular complex formation between berberine and p-sulfonated calixarenes, Chem. Phys. Lett. 424 (2006) 71-76.

[3] J.J. Inbaraj, B.M. Kukielczak, P. Bilski, S.L. Sandvik, C.F. Chignell, Photochemistry and photocytotoxicity of alkaloids from goldenseal (hydrastis canadensis l.) 1. Berberine, Chem. Res. Toxicol. 14 (2001) 1529-1534.
